# The relation between human hair follicle density and touch perception

**DOI:** 10.1038/s41598-017-02308-9

**Published:** 2017-05-31

**Authors:** Emma H. Jönsson, Johanna Bendas, Kerstin Weidner, Johan Wessberg, Håkan Olausson, Helena Backlund Wasling, Ilona Croy

**Affiliations:** 1 Institute of Neuroscience and Physiology, Sahlgrenska Academy, University of Gothenburg, Gothenburg, Sweden; 2 Department for Psychotherapy and Psychosomatic Medicine, Dresden University Hospital, Technische Universität Dresden, Dresden, Germany; 3 Department of Clinical and Experimental Medicine, Center for Social and Affective Neuroscience, Linköping University, Linköping, Sweden

## Abstract

Unmyelinated low threshold C-tactile fibers moderate pleasant aspects of touch. These fibers respond optimally to stroking stimulation of the skin with slow velocities (1–10 cm/s). Low threshold mechanoreceptors are arranged around hair follicles in rodent skin. If valid also in humans, hair follicle density (HFD) may relate to the perceived pleasantness of stroking tactile stimulation. We conducted two studies that examined the relation between HFD and affective touch perception in humans. In total, 138 healthy volunteers were stroked on the forearm and rated the pleasantness and intensity. Stimulation was performed by a robotic tactile stimulator delivering C-tactile optimal (1, 3, 10 cm/s) and non-optimal (0.1, 0.3, 30 cm/s) stroking velocities. Additionally, a measure of discriminative touch was applied in study 2. HFD of the same forearm was determined using the Cyanoacrylate Skin Stripping Method (CSSM), which we validated in a pretest. Women had higher HFD than men, which was explained by body size and weight. Furthermore, women rated affective touch stimuli as more pleasant and had higher tactile acuity. Depilation did not affect touch perception. A weak relationship was found between the C-tactile specific aspects of affective touch perception and HFD, and the hypothesis of HFD relating to pleasant aspects of stroking only received weak support.

## Introduction

The hair follicle is surrounded and innervated by a large network of sensory afferents^1^ and it has long been suggested that hair follicles are important for the sense of touch^2^. The aim of the current studies was to test whether hair follicle density (HFD) is related to the affective component of touch.

Hair follicles are present all over the body, except for the palms of the hands, the soles of the feet, the genitalia and the lip vermillion^3–6^. The adult human has three types of hair: terminal, vellus and intermediate hair. The terminal hairs are thick and pigmented and can be found in androgen-dependent areas (e.g. scalp, beard and axilla) and in some androgen-independent areas (such as the eyebrows). Vellus hairs are thin, short and unpigmented and cover the whole body, except for the glabrous skin. Intermediate hairs can be described as a mixture between the terminal and vellus hairs and are found together with the vellus hairs on the arms and legs of adults^1^. All hair follicles are developed early in the fetal period and no new hair follicles are formed after birth, implying that HFD in an adult will depend on how much that particular body part grows after the formation of the hair follicles^7^. This has two interesting consequences: First, it can be assumed that there is a certain inter-individual variation of HFD and this variation will be rather stable over time, with some degeneration being possible. Hence, a person with low HFD will remain a person with low HFD. Second, HFD in the adult depends on the degree of growth of particular body parts after the formation of the hair follicles^7^. Correspondingly, the density of hair follicles is highly variable over the human body, with highest densities in the face and lowest in the more distal parts of the body, such as the calf^6–9^.

Recent work in mouse hairy skin has shown that the hair follicles are innervated by C low threshold mechanoreceptors (C-LTMRs)^10, 11^. The terminal projections of C-LTMRs are only found in hairy skin and they appear to encircle and penetrate the hair follicle^11^. Furthermore, C-LTMRs selectively innervate certain types of hair follicle, namely the awl/auchene and zigzag, but not guard hair follicles^10^. Furthermore, the dorsal root ganglions (DRGs) innervating distal limbs contain fewer C-LTMRs than DRGs innervating proximal limbs^10^.

The human equivalent to the C-LTMRs are called C-tactile afferents. The C-tactile afferents, which exist exclusively in hairy skin, respond optimally to a slow, light stroking (1–10 cm/s) delivered at skin temperature. Stroking the skin at faster and slower velocities decreases the firing frequency. The characteristics of C-tactile optimal stroking correspond to a human-to-human caress^12–15^ and the firing frequency is correlated with subjective ratings of perceived pleasantness^13^. C-tactile afferents have also been suggested to be involved in detecting the erotic component of touch to non-genitalia skin^13, 16^. Taken together, the main role of these nerve fibers appears to be the moderation of the affective experience of touch^15^. It has been further shown that adults who are asked to apply a stroking caress to their infant or their partner, spontaneously use velocities that target C-tactile afferents^17^.

The density of C-tactile afferents appears to predict the perceived pleasantness of tactile stroking stimulation. In a group of rare patients suffering from a significant reduction of thin nerve fibers, including C-tactile fibers, ratings of perceived pleasantness were significantly reduced compared to healthy controls^18^. Other clinical disorders where altered C-tactile processing has been found include for example autism^19, 20^ and anorexia nervosa^21^.

The discriminative properties of touch on the other hand, such as object identification, are mainly processed by thick, fast conducting, myelinated Aβ afferents^22^. In contrast to C-tactile fibers, the firing frequency of Aβ afferents increases linearly with increasing velocity^13^. The firing frequency of Aβ afferents is correlated with ratings of touch intensity^13^.

One way to present the C-tactile specific experience of touch is by measuring *pleasant touch awareness*. This variable represents the C-tactile specific, mainly peripherally moderated experience of touch. It has been shown before, that pleasantness ratings to stroking stimulation show a strong correlation to the firing frequency of C-tactile fibers and that the C-tactile firing frequency follows an inverted U-shaped curve^13^. We therefore assume, that a high bending of the curve reflects the perception of C-tactile mediated aspects of touch. This bending is calculated as the rating difference of C-tactile optimal vs. sub-optimal stroking velocities, weighted by the overall touch ratings^19^. The *overall touch pleasantness* on the other hand represents a top down modulated experience of touch and is calculated as averaged ratings over all stroking velocities^19^. The stimulation of Aβ afferents in a stroking experiment is best captured by ratings of intensity^13^. Another measurement of Aβ signaling is the two-point discrimination threshold.

We hypothesized, that human hair follicles could be associated with C-tactile afferents in a similar way as shown in mice. Affective touch perception may depend on C-tactile fiber density and positively relate to HFD. The spatial summation of C-tactile input to the central nervous system due to higher HFD and C-tactile fiber density may thus be encoded as higher levels of pleasant touch awareness.

To examine the relationship between HFD and affective touch perception, we first defined the forearm as a body site representative for the overall HFD of the body using the cyanoacrylate skin stripping method (CSSM)^7^. Secondly, in a small subsample we tested whether depilation changed the perception of touch stimulation. These two pretests are presented in the methods section. We then tested HFD and the affective perception of slow C-tactile afferent targeting stroking, in order to determine a potential correlation. Using a robotic device validated for psychophysical examination of hedonic qualities of touch^23^, participants were stroked with C-tactile optimal (1, 3, 10 cm/s) as well as non-optimal (0.1, 0.3, 30 cm/s) velocities (compare previous studies^12, 24, 25^) and asked to rate the affective domains of pleasantness and eroticism. The relation between C-tactile afferent stimulation and non-genitalia directed erotic touch perception has been shown in a recent paper^16^. The ratings of eroticism are presented in the supplementary information.

According to previous work^19^, two main affective touch outcome variables were defined: The *pleasant touch awareness* and the *overall touch pleasantness*. In an additional study, we replicated and extended study 1. Ratings of intensity and two-point discrimination (study 2) were included in order to test for discriminative, Aβ mediated, touch. We also assessed gender differences in HFD, discriminative and affective touch perception.

## Results

### Moderators of hair follicle density

**Figure 1:**
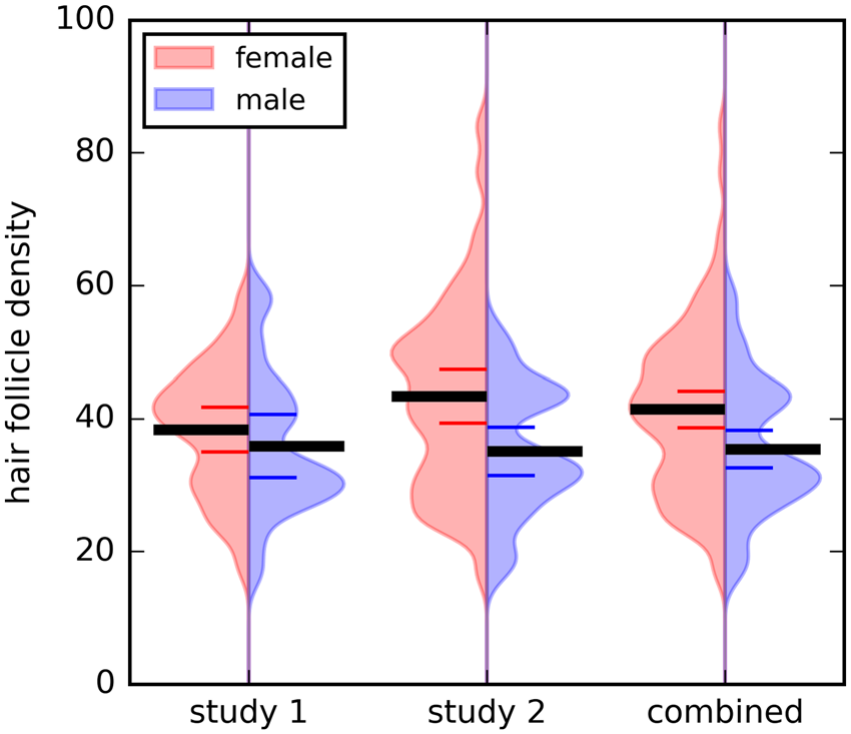
Hair follicle density of the forearm compared between men and women in study 1, study 2, and the combined sample. There was no significant difference in HFD between men and women in study 1. Women presented higher HFD on the forearm in study 2 and in the combined sample. However, this difference was explained by women’s smaller body size. On the x-axis the density of the HFD is plotted. The black line represents the statistical mean value, the red and blue lines, respectively, represent 95% confidence intervals.

In the **first study**, the HFD of the forearm skin was successfully calculated in 55 out of 58 participants samples, yielding a mean number of 37.4 ± 10.0 hair follicles per cm^2^ (range: 17–59). HFD was significantly negatively correlated to the weight of the participants (r = −0.303, p = 0.031) and further negatively related to the body height, however this correlation was not significant (r = −0.247, p = 0.1). Hence, the height and weight were included as covariates in the following analysis of gender differences. Although women displayed a higher HFD than men (women: N = 33, mean 38.39 ± 9.52; men: N = 22, mean 35.91 ± 10.77, compare Fig. 1), the difference was not significant after inclusion of weight and height in the analysis (F[51] = 0.36, p = 0.6).

The results of HFD calculations in study 1 were replicated in **study 2**. Here, HFD was successfully calculated in all 80 samples, displaying a mean number of hair follicles per cm^2^ of 40.38 ± 13.29 SD (range: 17–84) and HFD decreased with increasing volume of the participant’s body (correlation to height: r = −0.312, p = 0.005; to weight: r = −0.164, p = 0.2). There was a significant difference in HFD between men and women with women displaying a higher number of hair follicles per cm² compared to men (women: 43.37 ± 14.23/cm²; men: 35.1 ± 9.58/cm²). However, this difference was not significant after inclusion of weight and height as covariates in the analysis (F [79] = 1.3, p = 0.3; Fig. 1).

### Combined analysis

The datasets of the original and the replication study were combined in order to allow detection of subtle effects by an increased sample size of 135 participants in total. This was possible, as the participants of both studies did not differ significantly in gender distribution (p = 0.6), age (p = 0.1), weight (p = 0.8), height (p = 0.7), BMI (p = 0.3) or HFD (p = 0.2).

Over all participants, the HFD was significantly correlated to participants’ weight (r = −0.203, p = 0.021) and height (r = −0.286, p = 0.001). Again, women had a higher HFD (mean 41.4 ± 12.8 SD) than men (mean 35.5 ± 10 SD, compare Fig. 1) and again those differences were not significant after inclusion of weight and height as covariates (F[1, 129] = 0.43, p = 0.5).

### Perception of affective and discriminative touch

For analysis of the perception of affective and discriminative touch, different measurements can be distinguished: the general perception of affective touch is measured by the overall touch pleasantness, whereas C-tactile mediated affective touch perception is described by pleasant touch awareness (for calculation of these values, see Methods section). The discriminative touch perception mediated mainly by Aβ fibers was determined through the overall intensity ratings and, in study 2, the two-point discrimination threshold.

**Figure 2:**
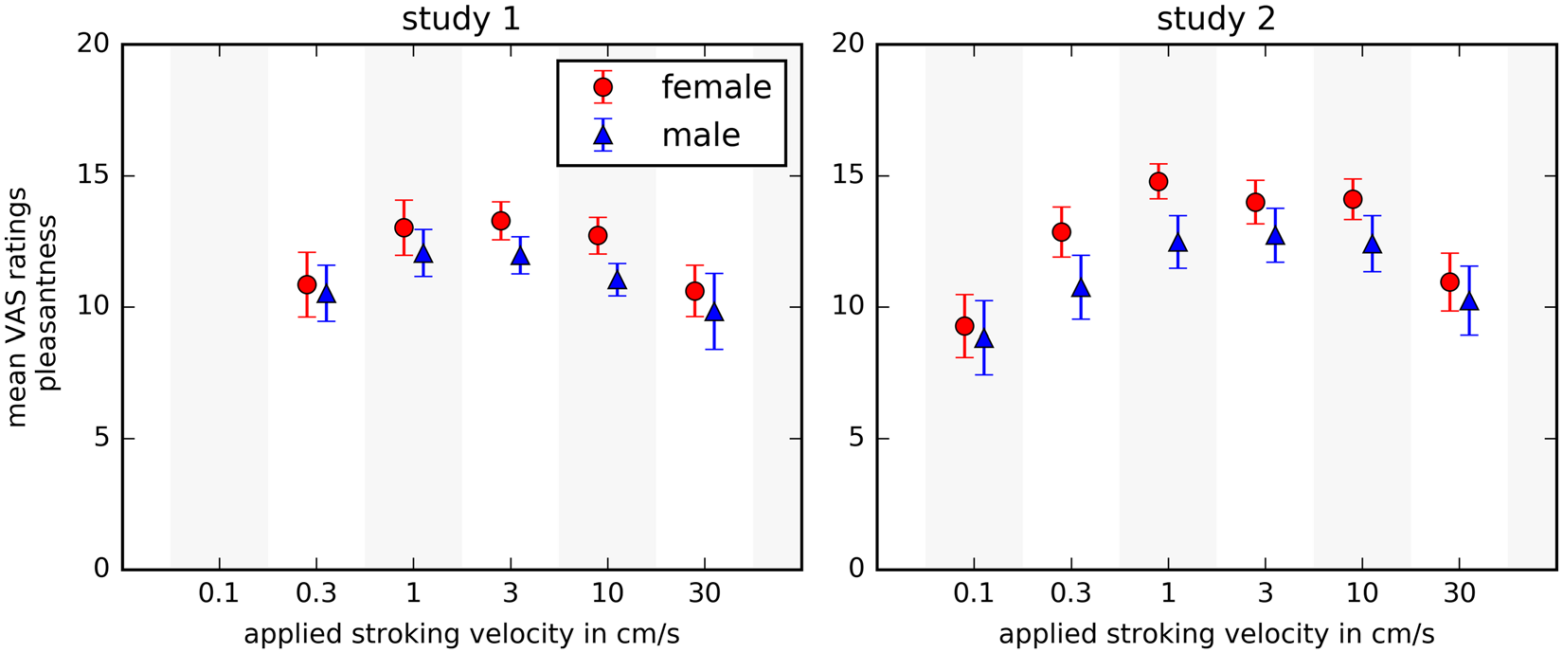
Gender-specific pleasant touch perception. Ratings of perceived pleasantness compared between men and women in study 1 and study 2. Female participants rated higher across all applied stroking velocities than male participants. The error bars represent 95% confidence intervals.

In the **first study**, pleasantness ratings of different stroking velocities followed an inverted U-shape peaking at 3 cm/s (main effect of velocity: F[2.7, 153.9] = 15.8, p < 0.001; compare Fig. 2). Further, there was a significant main effect of velocity on intensity ratings (F [2.3, 129.4] = 10.0, p < 0.001), where faster velocities were rated as more intense than slower velocities.

The overall touch pleasantness differed between male and female participants with women rating pleasantness of touch significantly higher than men (F[1, 55] = 4.7, p = 0.034). There was no significant interaction effect between gender and velocity (pleasantness: p = 0.5) and no significant gender difference on pleasant touch awareness (p = 0.8), meaning that the shape of the inverted U curve did not differ between genders. Women rated the touch slightly more intense than men, however, this difference was not significant (p = 0.1).

These results were replicated in **study 2**. Here, the touch pleasantness ratings followed a similar inverted U-shaped curve with the highest pleasantness ratings for 3 cm/s (main effect of velocity: F[3.01; 210.8] = 38.52, p < 0.001 compare Fig. 2). Again, there was also a significant main effect of velocity on intensity ratings (F[2.62, 180.67] = 31.96, p < 0.001) and faster velocities were rated as more intense than slower velocities.

**Figure 3:**
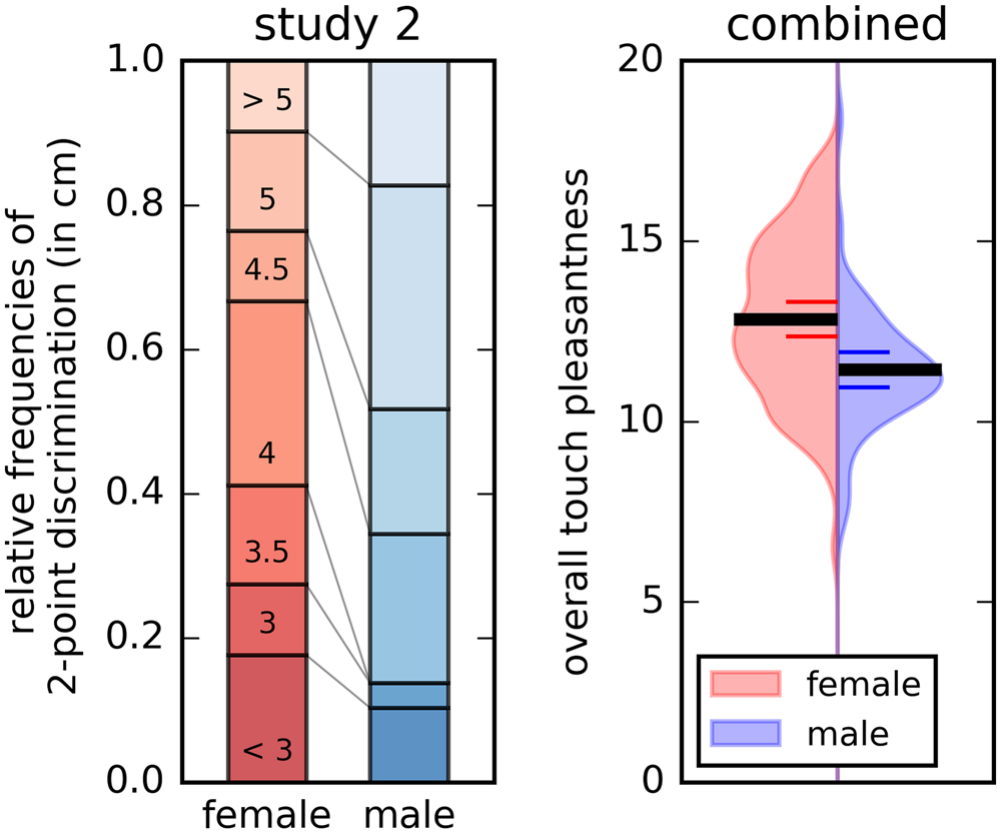
Two-point discrimination (study 2) and overall touch pleasantness (combined sample). Women present a lower two-point discrimination threshold then men. Touch pleasantness ratings were higher for women than men in the pooled data set (study 1 and 2). In the representation of overall touch pleasantness, the density is plotted on the x-axis. The black line represents the statistical mean value, the red and blue lines, respectively, represent 95% confidence intervals. The numbers and color codes on the bar representing the female values describe the distance of the two indenting stimuli in the course of the assessment.

The overall touch pleasantness differed significantly between men and women, with women rating the pleasantness of touch higher than men (F[1, 69] = 10.38, p = 0.002). Again, there was no significant interaction effect between gender and velocity and no significant gender difference on pleasant touch awareness (p = 0.9), indicating that the shape of the inverted U curve did not differ between the genders. The overall touch intensity was higher for women than for men (F[1, 68) = 11.36, p = 0.001). The median two-point discrimination threshold was 4 (25^th^ to 75^th^ percentile: 3.5 to 5) and women had a lower threshold, (median 4; 25^th^ to 75^th^ percentile: 3–4.5) than men (median 4.5; 25^th^ to 75^th^ percentile: 4 to 5; Z = 2.6, p = 0.009, compare Fig. 3).

### Combined analysis

Comparison between the studies revealed a significant difference in the overall touch pleasantness (t [134] = 3.05, p = 0.003) with ratings being higher in the sample of study 2. Ratings for pleasant touch awareness and for overall touch intensity did not differ between the sample groups (pleasant touch awareness: p = 0.72; overall touch intensity: p = 0.64).

Over all participants, the significant effect of gender on touch perception remained stable, with women rating pleasantness and the intensity of touch higher than men (pleasantness: F[1, 135] = 15.0, p < 0.001, intensity: F[1, 135] = 16.0, p < 0.001). Furthermore, overall touch pleasantness and overall touch intensity differed significantly between men and women (p < 0.001, compare Fig. 3 for overall touch pleasantness). There was no interaction effect between gender and velocity on the ratings (pleasantness: p = 0.4). In line, there were no significant gender differences in pleasant touch awareness (p = 0.8), indicating that the shape of the inverted U curve did not differ between the genders.

### Perception of touch in relation to HFD

In **study 1**, there was no significant correlation between HFD and pleasant touch perception (overall touch pleasantness: r = 0.079, p = 0.6; pleasant touch awareness: r = 0.028, p = 0.8). Furthermore, there was no correlation between intensity ratings and HFD (overall touch intensity: r = 0.180, p = 0.2). Inclusion of height and weight as control variables resulted in no changes of the correlations.

**Figure 4:**
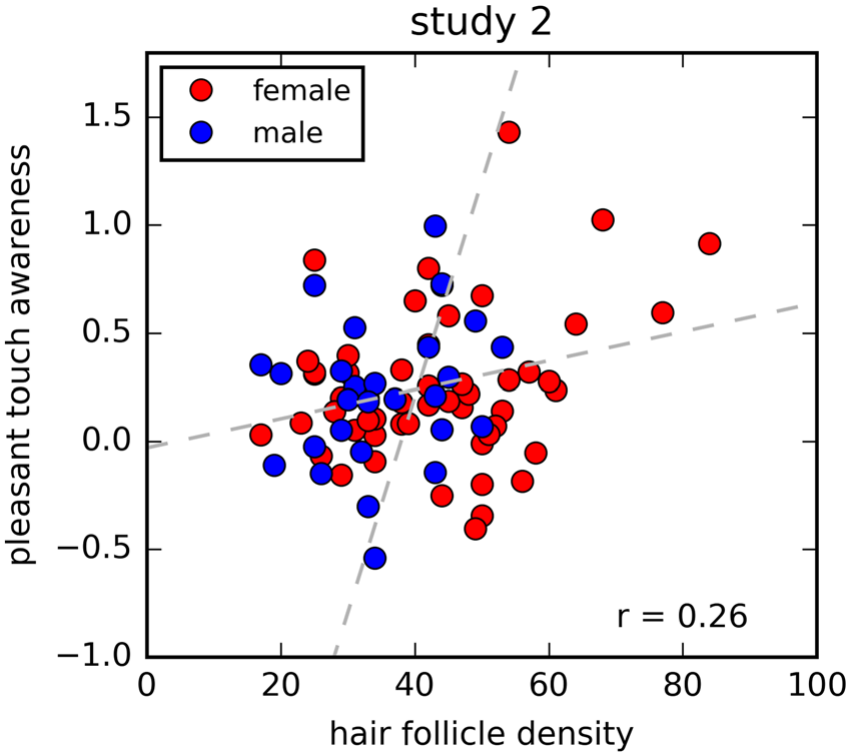
Affective touch perception in relation to HFD in study 2. There was a positive correlation between pleasant touch awareness and HFD in study 2 across all participants. The dotted lines represent the two regression lines obtained by regressing pleasant touch awareness on hair follicle density and vice versa. The depicted r represents the Pearson correlation coefficient.

The result from **study 1** was mainly replicated in **study 2**. Again, HFD was not related to overall pleasantness (r = −0.112, p = 0.322) or overall touch intensity (r = 0.039, p = 0.746) and there was no significant correlation between HFD and the two-point discrimination threshold (r = 0.027, p = 0.8). However, HFD correlated positively to pleasant touch awareness (r = 0.269, p = 0.016, compare Fig. 4), implying that a high pleasantness rating to C-tactile specific stimulation was related to higher HFD. Inclusion of weight and height as control variables did not change the results: the correlation between HFD and pleasant touch awareness remained stable (r = 0.261, p = 0.024), all other correlations were non-significant.

### Combined analysis

Combining the data from both studies revealed no significant correlations between the HFD and measures of touch (overall touch pleasantness: r = −0.012, p = 0.9; pleasant touch awareness: r = 0.134, p = 0.1; overall touch intensity: r = 0.096, p = 0.3). Inclusion of height and weight as control variables did not change the results.

## Discussion

The aim of the present studies was to investigate a potential relationship between HFD on the human forearm and affective touch perception from stimulation to the same body site. However, a relation between HFD and affective touch perception was only found for study 2, where pleasant touch awareness was moderately correlated with HFD. Interestingly, this result was neither found in study 1, nor in the overall sample. Previous studies have found C-LTMRs to be distributed around hair follicles^10, 11^, leading to our hypothesis that C-tactile afferents are associated to hair follicles in a similar way in humans and that this influences the perception of stroking touch stimulation. We only found weak support for this hypothesis. Several aspects should be considered here: Li and colleagues used genetic labeling to visualize the distribution of nerve fibers in rodent skin. In comparison, our approach was less specific and relied on the assumption that differences in C-tactile fiber density are reflected in affective touch perception. Although it has been shown that pleasant touch perception relates to C fiber density^18^, it is not clear, how C fiber density impacts affective touch perception specifically. We assumed this relation to be linear with a higher density of C-tactile fibers resulting in higher ratings of perceived pleasantness, but a staircase model is likewise conceivable. Hence, the C-tactile density may not be important for affective touch perception as long as a critical amount of intact C-tactile fibers are present. In our sample of healthy people, it is very likely that such a critical amount would be conserved in all participants. On the other hand, disturbances in affective touch perception have been found in patients suffering from anorexia nervosa^21^. These patients have a very low body volume and, following the argument that all hair follicles are formed in utero, should have more densely packed hair follicles and thereby also higher density of C-tactile afferents. However, in these patients the potentially higher density of C-tactile afferents does not increase the pleasantness ratings. On the contrary, the ratings are decreased compared to normal participants^21^.

Our hair follicle density measurement was based on skin sample counting. Although we made every effort to ensure reliability of this method and our findings are in very good alignment with previous results^7^, some questions remain unanswered. For instance the hair shaft length and even type of hair growing in the follicle can vary with the hair follicle cycle and is influenced by internal and external factors such as hormonal levels or lifestyle^26, 27^. This may impact the visibility of the hair follicles under the microscope. Another limitation of the study is the conduction of the experiment on one body site only, the forearm. However, this site is representative for pleasant touch perception^28^ and seemingly represented the individual HFD well.

Taken together, HFD in healthy subjects does not strongly relate to human affective touch perception and, as far as we tested this, it neither relates to human discriminative touch perception. However, the study revealed more interesting results, which are worth further discussion.

The presented results describe the determination of the forearm as a body site representing the overall **hair follicle density** of the body and reproduce findings on the distribution of hair follicles across the body^7^. To the best of our knowledge, gender differences in HFD have not been reported before. Women are typically smaller than men^29^, which is confirmed in our sample. Differences in body size should matter for HFD as hair follicles are formed in utero^30^ and the HFD in the adult is dependent on the growth of the different body parts. Indeed, we found a negative correlation between HFD and weight of the participants in study 1 as well as a negative correlation between HFD and height in study 2. Over the whole sample, both height and weight significantly related to HFD. The higher HFD in women was thus fully explained by gender differences in body size.

For **affective touch perception**, our results confirmed the previously reported inverted U-shape of the ratings for pleasantness (e.g. refs 12, 16, 22 and 24). The differences of the rating levels between study one and two are most likely explained by slight methodological differences (anchors of visual analogue scales and application of an additional velocity in study 2).

Women rated the stroking stimulation as more pleasant, irrespective of whether touch was applied at C-tactile optimal or suboptimal velocities. This effect has been observed in previous work^23^. Further, women rated all stroking stimulations as more intense and had a lower two-point discrimination threshold. This suggests that women are more sensitive to affective and discriminative aspects of touch, however no consistent gender differences were found for the shape of the curve (measured as pleasant touch awareness) which describes C-tactile specific perception.

The gender differences could potentially be explained by differences in peripheral nerve density, top-down processing or a combination of the two. It is worth noting that affective touch on the peripheral level is mediated by input of both C-tactile and Aβ afferents^22^. The lack of a difference in pleasant touch awareness suggests that the proportion of peripheral C-tactile input is similar for both sexes. The differences in discriminative sensitivity suggest a higher density of Aβ afferents in women. Obviously, this is an oversimplification since spinal cord and brain processing play a substantial role for the tactile experience. A similar gender effect on discriminative touch perception on the fingertips was previously reported and related to the size of the fingertips^31^. The potentially higher afferent input in women may explain the gender specific affective touch rating differences: such a higher input may not only increase the perceived intensity of stroking but also the pleasantness by making the tactile stimulation more perceivable. In addition, top-down influences may mediate the gender effects of affective touch perception. In general, top down processing has been shown to have a modulatory effect on the perception of touch^32^. Potential factors of bias, such as effects of expectancy, effects of the investigator or interpretation of the scales^33^ might differ between genders and thereby affect touch perception. All data collection was done by female researchers which potentially influenced the rating behavior of men and women differently, something that has been seen in rating behavior of pain previously^34^. For the ratings, individuals could have had different concepts of what the terms pleasant or intense entail; potentially this could be gender specific and to some degree explain the differences in ratings.

## Conclusion

Extrapolating from the findings by Li *et al*.^10^ where C-tactile fibers where found to be located around specific types of hair follicles in mice, we formed the hypothesis that the number of hair follicles would indicate the underlying C-tactile afferent density in human hairy skin and this would in turn affect the perception of affective touch. Only weak support for this hypothesis was found. However, important findings of gender differences in ratings of touch perception were made. These factors should be considered in future studies exploring affective touch in humans.

## Methods

### Participants

Fifty-eight participants took part in **study 1** (34 female; mean age 26.2 years ± 6.3 years SD, range 19–51 years; for more demographic data compare Table 1). Most of these participants (N = 47) were also included in a previous study^14^. Inclusion criteria were subjectively good health, good knowledge of the Swedish language and age above 18 years. Exclusion criteria were any skin condition, impairments of the sense of touch or sight, and allergic reaction to cyanoacrylate. 11 of the participants also participated in a small study on the effect of hair on the perception of touch.

**Table 1:** Gender-specific demographic features of the sample groups of study 1 and 2 and the combined sample.

		Whole sample (N=58) Mean ± SD	Women (N = 34) Mean ± SD	Men (N = 24) Mean ± SD	t-test for gender differences (significant results printed bold)
Study 1	Height (cm)	174.1 ± 10.4	167 ± 6.8	183 ± 6.9	**t[54] = −8.5, p < 0.001**
	Weight (kg)	67.5 ± 11.3	61.4 ± 8.1	76.4 ± 9.3	**t[52] = −6.3, p < 0.001**
	BMI (kg/m²)	22.3 ± 2.1	21.9 ± 2.0	22.9 ± 2.1	t[52] = −1.9, p = 0.067
	HFD	37.4 ± 10	38.4 ± 9.5	35.9 ± 10.2	t[53] = 0.9, p = 0.37
	Age (years)	26.2 ± 6.3	26.7 ± 6.6	25.5 ± 5.7	p > 0.1
		Whole sample (N = 80)	Women (N = 51)	Men (N = 29)	t-test
Study 2	Height (cm)	173.4 ± 9.6	168.5 ± 6.97	182.1 ± 6.99	**t[78] = −8.44, p < 0.001**
	Weight (kg)	69.4 ± 18.3	62.2 ± 10.5	81.8 ± 22	**t[77] = −5.34, p < 0.001**
	BMI (kg/m²)	22.9 ± 4.9	21.9 ± 3.7	24.6 ± 6.1	**t[77] = −2.45, p = 0.017**
	HFD	40.4 ± 13.3	43.4 ± 14.2	35.1 ± 9.58	**t[78] = 2.79, p = 0.007**
	Age (years)	24.9 ± 4.1	24.2 ± 3.9	26.1 ± 4.3	p > 0.05
		Whole sample (N = 138)	Women (N = 85)	Men (N = 53)	t-test
Combined sample	Height (cm)	173.7 ± 9.9	168.0 ± 6.9	162.6 ± 6.9	**t[134] = 12.01, p < 0.001**
	Weight (kg)	67.8 ± 12.1	61.9 ± 9.6	77.3 ± 9.2	**t[131] = 9.08, p < 0.001**
	BMI (kg/m²)	22.7 ± 4.0	21.9 ± 3.1	23.9 ± 4.8	**t[131] = 2.87, p = 0.005**
	HFD	39.2 ± 12.1	41.4 ± 12.8	35.5 ± 10.0	**t[133] = 2.85, p = 0.005**
	Age (years)	25.4 ± 5.2	25.2 ± 5.3	25.8 ± 5.0	p > 0.1

Eighty participants took part in the replication study - **study 2** (51 female, mean age 24.86 ± 4.1 years SD, range 18–36 years; for more demographic data compare Table 1). Inclusion criteria were subjectively good health, good knowledge of the German language and age between 18 and 40 years. Exclusion criteria were any skin condition, impairments of the sense of touch or sight, and allergic reaction to cyanoacrylate.

All participants received financial reimbursement for their participation. Ethical approval was obtained from the central ethics committee of the University of Gothenburg in Gothenburg, Sweden (**study 1**) as well as the ethics committee of Dresden University of Technology (**study 2**), respectively. The studies were performed according to the Declaration of Helsinki on Biomedical Research Involving Human Subjects. All participants received information about the study prior to signing informed consent.

### Tactile stimulation and psychophysical ratings

For **study 1**, the participants were seated in a comfortable chair in front of a computer screen. Stroking tactile stimulation was applied to the left (N = 47) or to the right dorsal forearm (N = 11). The stimuli were delivered by a soft brush (6 cm wide paintbrush made of soft goat’s hair) attached to a robotic device (Rotary Tactile Stimulator, RTS, Dancer design; St Helens, UK). Stimuli were applied over a distance of 6.5 cm with a force of 0.4 N using five different stroking velocities: 0.3, 1, 3, 10 and 30 cm/s. The intermediate velocities (1, 3 and 10 cm/s) activate C-tactile afferents optimally while faster and slower velocities 0.3 and 30 cm/s activate C-tactile afferents sub-optimally. Each velocity was repeated three times in a pseudorandomized order, such that no two subsequent stimulations were of the same velocity. To remove environmental disturbances, the participants wore headphones playing pink noise at a comfortable volume. They also wore visually shielding goggles to prevent them from seeing the stimulation.

After each brush stroke the participant was asked to rate the stimulus on visual analog scales (VAS) that appeared on the computer screen. For each stimulus three properties were rated: pleasantness (anchors: extremely unpleasant (−10) and extremely pleasant (10)), intensity (anchors: not at all intense (0) and extremely intense (20)) and eroticism (see supplementary data). The participant indicated his/her VAS rating using a computer mouse positioned within easy reach on the non-stimulated side. The ratings were automatically translated into numerical values. The participants were ensured that the rating procedure would not be supervised.

In **study 2**, the participants first received the same tactile stimulation on the left forearm as described above for study 1, with the exception that six velocities (0.1, 0.3, 1, 3, 10 and 30 cm/s) were administered instead of five. As in study 1 after each brush stroke the participants rated the perceived pleasantness and intensity on VAS with the anchor points “not at all pleasant/intense (0)” and “extremely pleasant/intense (20)”, respectively.

Next, on the same site on the forearm, the two-point discrimination threshold was determined using a metal devices with two tips (alike a usual divider) in variable distances (3 cm, 3.5 cm, 4 cm, 4.5 cm, 5 cm). The range of probes was guided by a previous study^35^. The participants were asked to close their eyes and were stimulated with a slight, gentle indentation of both ends of the device on the central dorsal forearm. They were asked to report whether they perceived one or two indentations. The examiner carefully touched the skin with the two tips of the device simultaneously. The devices were applied in decreasing order, starting with 5 cm distance until the participants perceived the two indentations as one in at least one out of five stimulations. The last distance that was recognized correctly in five out of five trials was considered the two-point discrimination threshold.

### HFD

After the psychophysical experiment a skin sample from the stimulated part of the forearm was obtained using the Cyanoacrylate Skin Stripping Method (CSSM, compare^7^). Therefore, an area of 2 × 2 cm was marked and shaved before applying one drop of cyanoacrylate adhesive (Loctite precision; Henkel, Düsseldorf, Germany) onto the skin and covered with a glass slide. The glass slide was gently pressed against the skin so that a thin film of glue was formed. After about 30 seconds the glass was lifted off the skin. The number of hair follicles in 1 cm^2^ was counted in each skin sample using a light microscope. All samples were anonymized and counted by the same person (EHJ for study 1, JB for study 2).

A pretest was conducted for determination of the best site for HFD determination. Fifteen participants took part in this pretest (8 female, mean age 23.9 ± 3.0 years SD, range 20–32 years), who received financial reimbursement. Good subjective health served as inclusion criteria, whereas any skin condition and allergic reaction against cyanoacrylate were used as exclusion criteria. Ethical approval was obtained from the ethics committee of Dresden University of Technology. Hair follicle samples were collected on 9 different sites of the body - forehead, neck, chest, lower abdomen, lower back, upper arm, forearm, thigh and calf as performed in an analogical study by Otberg and colleagues^7^. The HFD per body site and the overall HFD, calculated as the mean value of the 9 different sampled sites, were obtained and z-standardized for further investigation in order to account for the distribution of hair follicle densities between body sites. In result, the obtained HFD for the different body sites agreed with the densities found by Otberg *et al*.^7^, and the densities decreased in a proximal-distal fashion, with the highest densities found on the forehead and the lowest on the calf (compare Table 2). The relationship between the standardized hair follicle densities across body sites was carried out using Pearson correlations. Overall HFD correlated most strongly to the HFD of the forearm (r = 0.830, p < 0.001). Hence, HFD of the forearm was related to HFD of other body parts (forearm- thigh: r = 0.808, p < 0.001; forearm- calf: r = 0.671, p = 0.009; forearm-upper arm: r = 0.746, p = 0.001; forearm- chest: r = 0.621, p = 0.013, compare Table 2). However, no significant correlations could be observed between the HFD of the forearm and those of forehead, neck, abdomen or lower back.

**Table 2:** Hair follicle densities at the 9 sampled body sites in pretest (N = 15), their correlation with the overall mean density and with each other.

Body site	Hair follicles per cm² (N = 15) Mean ± SD	Correlations (significant results printed bold)									
		Overall HFD	Forehead	Neck	Chest	Upper arm	Forearm	Back	Abdomen	Thigh	Calf
Forehead	285.0 ± 84.1	r = 0.465 p = 0.08	—								
Neck	47.3 ± 17.1	r = 0.130 p = 0.643	r = −0.083 p = 0.769	—							
Chest	28.4 ± 6.4	**r = 0.667 p = 0.007**	r = 0.289 p = 0.296	r = −0.204 p = 0.466	—						
Upper arm	45.9 ± 14.5	**r = 0.781 p = 0.001**	r = 0.232 p = 0.405	r = −0.185 p = 0.509	**r = 0.540 p = 0.038**	—					
Forearm	40.3 ± 14.9	**r = 0.840 p < 0.001**	r = 0.364 p = 0.183	r = −0.239 p = 0.391	**r = 0.621 p = 0.013**	**r = 0.746 p = 0.001**	—				
Back	24.7 ± 5.9	r = 0.286 p = 0.301	r = −0.172 p = 0.539	r = 0.382 p = 0.160	r = 0.244 p = 0.380	r = 0.053 p = 0.853	r = −0.035 p = 0.901	—			
Abdomen	16.9 ± 6.2	r = 0.354 p = 0.195	r = 0.061 p = 0.830	r = 0.151 p = 0.590	r = −0.053 p = 0.852	r = 0.261 p = 0.348	r = 0.057 p = 0.841	r = 0.482 p = 0.069	—		
Thigh	21.0 ± 8.0	**r = 0.678 p = 0.006**	r = 0.156 p = 0.579	r = 0.043 p = 0.878	r = 0.342 p = 0.212	**r = 0.668 p = 0.005**	**r = 0.808 p < 0.001**	r = −0.236 p = 0.398	r = −0.142 p = 0.614	—	
Calf	15.6 ± 3.6	**r = 0.559 p = 0.03**	r = 0.369 p = 0.176	r = −0.246 p = 0.378	r = 0.394 p = 0.146	r = 0.385 p = 0.157	**r = 0.67 p = 0.006**	r = −0.355 p = 0.195	r = −0.131 p = 0.643	**r = 0.566 p = 0.028**	—

We further confirmed reliability of the CSSM by assessing interrater reliability in 25 samples, including samples from the pre-test (see below, N = 5) and from study 2 (N = 20). Those were analyzed independently by JB and EHJ and the interrater reliability was calculated to 0.904.

### The effect of hair on the perception of touch

In a second pre-test, the effect of the hair on the perception of touch was assessed. The hair on the left forearm was chemically removed using hair removal crème (Veet; Reckitt Benckiser, UK) in eleven participants (7 female, mean age = 25.182 ± 3.710 SD, range 19–30 years). Those participants are also included in study 1. The tactile stimulation (as described for study 1) was then applied to both arms, in randomized order. The participants rated the perceived eroticism, pleasantness and intensity of the stimulation. The effect of the hair on the ratings of touch perception was tested using 2-way ANOVAs with velocity and hair/depilated as within subject factors. The results showed that there was no significant main effect of hair on any of the ratings (eroticism: F[1, 10] = 0.245, p = 0.631, pleasantness: F[1, 10] = 2.053, p = 0.182, intensity: F[1, 10] = 1.002, p = 0.341). Furthermore, there were no significant interaction effects (hair by and velocity) on the ratings (eroticism: F[2.179, 21.789] = 0.219, p = 0.823 (Greenhouse-Geisser corrected), pleasantness: F[4, 40] = 0.227, p = 0.921, intensity: F[4, 40] = 1.594, p = 0.195).

### Statistical Analysis

For both studies, statistical analysis was performed using IBM SPSS Statistics for Windows, Version 22.0 (IBM; Armonk, NY, USA). Demographic variables (age, weight, height and BMI) were compared between genders using t-tests.

**HFD** was correlated to the weight and height of the participants. Further the HFD was compared between genders using a univariate analysis of variance. We included the weight and height as covariates in this model in order to control for the relation between HFD and gender.

For **analysis of the touch ratings**, the average value for every skin stroking velocity was determined from the 3 repetitions. Three repeated measures ANOVAs, with velocities (5) as within subject factors and gender (2) as between subject factor were used to analyze the ratings on pleasantness and intensity. Greenhouse-Geisser corrected in order to account for violations of sphericity.

Two main affective touch outcome variables were defined: The *overall touch pleasantness/intensity* was calculated as the mean of all touch pleasantness/intensity ratings. The *pleasant touch awareness* reflects the highest C-tactile specific pleasantness ratings and is calculated as the difference of pleasantness ratings between C-tactile optimized (3 cm/s) and non-optimized stroking (30 cm/s), weighted by the overall touch pleasantness. This calculation derived from the largest difference in ratings between C-tactile and non-C-tactile targeted touch experienced in previous studies^13, 16^.

$$pleasant\,touch\,awareness=\frac{pleasantness\,rating\,at\,3\,\frac{cm}{s}-pleasantness\,rating\,at\,30\,\frac{cm}{s}}{overall\,touch\,pleasantness}$$

The relation between HFD and *pleasant touch awareness* and *overall touch* measures was tested using non-parametric correlations. The results of the eroticism ratings are available in the supplement. A potentially moderating effect of the weight and height was taken into account in an additional analysis. Therefore, a partial correlation between HFD and all touch variables was computed with the weight and height as control variable.

In **study 2**, the two-point discrimination threshold was additionally assessed in a non-parametric way and hence related to the participants’ sex and weight by Spearman correlation coefficient. Further, this threshold was compared between male and female participants using the Mann-Whitney U test and sex and weight were included in the gender-effect analysis by using general equation modelling (GEE), implemented in SPSS. The correlation between two-point discrimination threshold and HFD was further tested using Spearman correlation.

### Combined Analysis of Study 1 and 2

To account for different anchor points used in the separate studies, ratings for perceived pleasantness were re-scaled to the scale used in study 2 using a linear transformation (10 rating points were added to ratings of pleasantness obtained in study 1 to transform from −10/10 to 0/20 scale).

First, the participants of both studies were compared according to gender distribution (Chi Square test) and age, weight and height (t-test), as well as according to all outcome variables (HFD, pleasant touch awareness, erotic touch awareness, overall pleasantness, overall eroticism, overall intensity; all with t-test).

HFD was correlated to the weight and height of the participants. The effect of gender on HFD was computed using a univariate analysis of variance with the weight and height as a covariate. HFD was further correlated to all touch perception variables, as reported for study 1.

## Electronic supplementary material

Supplementary Information
