# Supplementary material for: The relation between human hair follicle density and touch perception
Source: Sci Rep. 2017 May 31;7:2499. doi: 10.1038/s41598-017-02308-9 (PMC5451466; doi:10.1038/s41598-017-02308-9)
Supplement: Supplementary file 1 — Supplementary Information [file 41598_2017_2308_MOESM1_ESM.pdf]

The relation between human hair follicle density and touch perception

Emma H Jönsson, Johanna Bendas, Kerstin Weidner, Johan Wessberg, Håkan Olausson, Helena Backlund Wasling, Ilona Croy

Supplementary Information

Background and Methods

Recent work has suggested an involvement of C-tactile afferent stimulation in non-genitalia directed erotic touch perception [16]. At this, our protocol of tactile stimulation additionally involved the determination of erotic touch perception as a quality of affective touch. Therefore, participants were equally asked to rate the applied stroking stimuli concerning the perceived eroticism on VAS from 0 (not at all erotic) to 20 (extremely erotic), as described in the Methods section. *Overall touch eroticism* was accordingly calculated as the mean of all touch eroticism ratings. *Erotic touch awareness* was computed similarly to pleasant touch awareness by subtracting the ratings of 30 cm/s from the ratings of 1 cm/s (which was experienced as the most erotic stimulus in a previous study [16]) and then dividing by the overall touch eroticism.

$$erotic\ touch\ awareness = \frac{eroticism\ rating\ at\ 1\ \frac{cm}{s} - eroticism\ rating\ at\ 30\ \frac{cm}{s}}{overall\ touch\ eroticism}$$

The relation between HFD and *erotic touch awareness* and *overall touch eroticism* was tested using non-parametric correlations.

Results: Erotic touch perception

In **study 1** the ratings of eroticism followed an inverted U-shape, peaking at 1 cm/s (main effect of velocity eroticism: $F[2.8, 155.6]=16.3$, $p < 0.001$, compare figure S1). The ratings differed between male and female participants with women rating the eroticism of touch significantly higher than men (eroticism: $F\ [1, 55] = 5.2$, $p= 0.026$). However, the shape of the inverted U curve did not differ between the genders and hence there was no significant interaction effect between gender and velocity ($p=0.4$), and no significant gender difference on erotic touch awareness ($p=0.5$)

These results were replicated in **study 2.** Here, the touch ratings followed a very similar inverted U-shaped curve with the highest eroticism ratings for 1 cm/s (main effect of velocity: $F[3.07, 215.02]= 41.59$, $p<0.001$, compare figure S1). Women rated the stroking touch stimulation more erotic ($F[1, 69]=9.99$, $p=0.002$) than men. There was no significant interaction effect between gender and velocity

(eroticism: p=0.3) and no significant gender difference in erotic touch awareness (p=0.9), indicating that the shape of the curve did not differ.

Combined analysis: Comparison between the studies revealed a significant difference in overall touch eroticism (t [135] = 2.78, p = 0.006), with ratings being higher in the German sample (replication study). Erotic touch awareness did not differ between the sample groups.

Over all participants, the significant effect of gender on touch perception remained stable, with women rating the eroticism of touch higher than men (F [1, 135] = 16.0, p<0.001, compare figure S2).There was no interaction effect between gender and velocity on the ratings (eroticism: p=0.4) and no significant gender differences in erotic touch awareness (p= 0.6).

Results: Perception of erotic touch in relation to HFD

In **study 1**, there was no significant correlation between HFD and overall touch eroticism: r = 0.161, p = 0.2 or erotic touch awareness: r = 0.073, p = 0.6. Inclusion of length and weight as control variables resulted in no major changes of the correlations.

This result was replicated in **study 2**. Again, HFD was not related to overall eroticism (r=0.006, p=0.957) or erotic touch awareness (r=0.138, p=0.222).

Combined analysis: No significant correlations were found between the HFD and overall eroticism: r = 0.100, p = 0.3 or erotic touch awareness: r = 0.006, p= 0.9. Inclusion of length and weight as control variables did not change the results.

Conclusion

Our results confirm a contribution of C-tactile afferents in the perception of erotic touch with C-tactile optimal stroking stimuli being perceived as the most erotic stimuli. In two studies conducted separately in two countries, a difference in the course of ratings across the applied stroking velocities between perceived pleasantness and eroticism was detected. Hereby, perceived eroticism peaks at lower velocities (1 cm/s) than pleasantness (3 cm/s) which suggests eroticism to present a distinct aspect of affective touch perception.

Figures

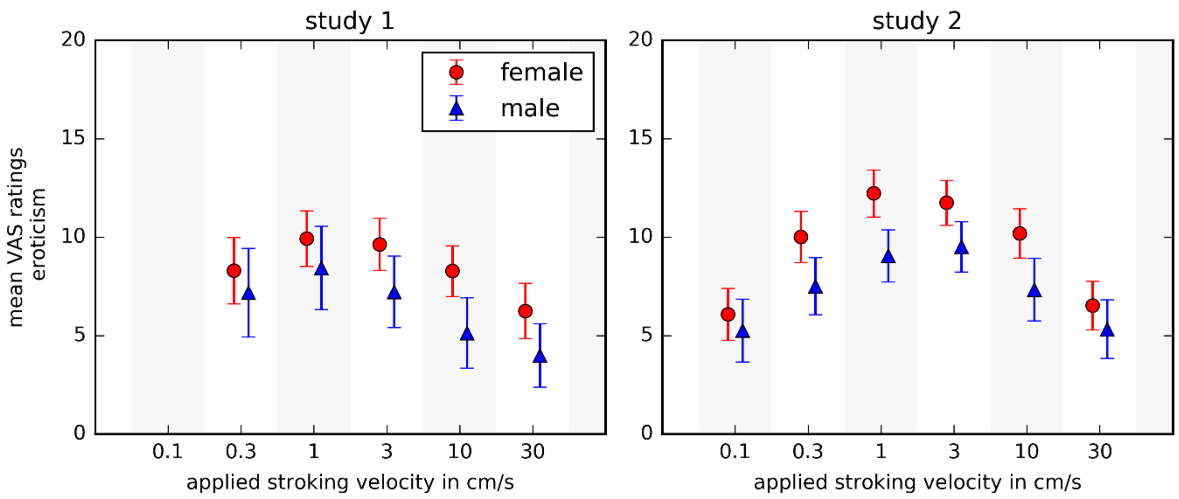

Figure S1 (Supplement) Gender-specific erotic touch perception.

Ratings of perceived eroticism compared between men and women in study 1 and study 2. Female participants present higher ratings across all applied stroking velocities than male participants. The error bars represent 95% confidence intervals.

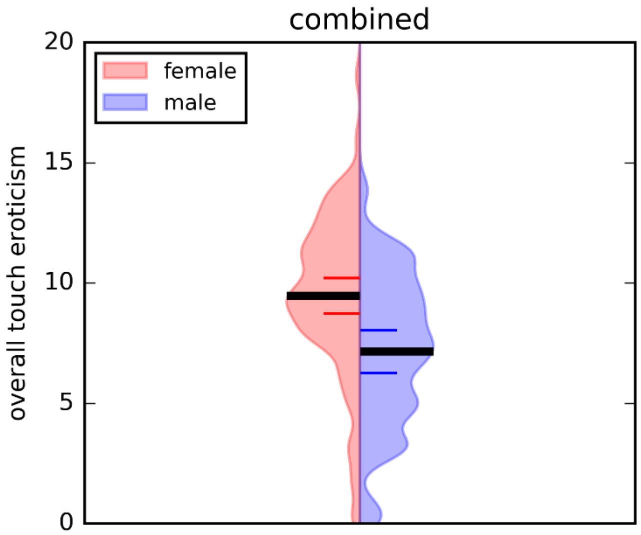

Figure S2 (Supplement) Overall touch eroticism in the combined sample.

Levels of overall touch eroticism compared between men and women for the pooled data set reveal higher levels of perceived eroticism in female participants. The density of overall touch eroticism is plotted on the x-axis. The black line represents the statistical mean value, the red and blue lines, respectively, represent 95% confidence intervals.
